# Book Reviews

**Published:** 1802-11-01

**Authors:** 


### CRITICAL ANALYSIS

OF THE

### RECENT PUBLICATIONS

ON THE DIFFERENT BRANCHES OF

### PHYSIC, SURGERY, & MEDICAL PHILOSOPHY.

---

*Obfervations on Pulmonary Confumption, or an Effay on the Lichen Iflandicus, confidered both as an Aliment and a Medicine in that Diforder, illuftrated by a coloured Engraving. By* J. B. Regnault, *late Phyfician to the Military Hofpitals and Forces of France, &c. London,* 1802, 8*vo. pp.* 82.

All fubftances taken into the human ftomach, may be confidered as food, medicine, poifon, or inert matters; and thefe denominations depend more on the *quantities* or dofes, than on the *qualities* of the fubftances themfelves. We do not, however, wifh to extend this pofition fo far, as to intimate that we believe arfenic may be fo dofed as to become a nutritious food, or water gruel a deadly poifon. What we wifh to ftate, is, that in the fcience of medicine, *quality* has been more attended to than it merited, and *quantity* almoft entirely overlooked. This accufation could not, with juftice, be applied to phyficians of the two or three preceding centuries, for they feldom prefcribed a draught or potion of fewer than fix or eight ounces, and often double that quantity. But modern refinement, luxury, and quackery, have very generally induced a belief, that difeafes may be cured by dofes of grains or ounces, by fyrups or watering places, by fleecy hofiery or tepid baths. Nay, nothing is more commonly faid by fqueamifh patients, than that they cannot take medicine, or fubmit to regimen; and yet they expect to be cured.

In thefe times, when the very mention of rhubarb, opium, or mercury to a patient is fufficient to prevent their being taken, nothing could have been more injurious than the publifhing the compofition of all the principal formulæ in Englifh.

Every practitioner of experience muft have obferved the great difference that arifes between the treatment of a patient who has been bred to the profeffion, and one who never heard the name of a medicine before his prefent illnefs: he muft have obferved and lamented the caufe of this difference. The late juftly-eminent Dr. Warren, in his laft illnefs, told Sir George Baker, who attended him, that *medicine was of no ufe to him.* Now this particular obfervation of a very great phyfician, is often made by perfons totally ignorant of medical fcience, who yet imagine they are capable of judging judging of the effects of medicines on their own conftitutions. If a patient has confidence in his phyfician, he fhould yield him implicit obedience; if not, he fhould fend for another. Nothing can be more unjuft than to make a medical practitioner refponfible for the event of a difeafe of which he had not the entire direction of the treatment; and yet nothing is more common than this fpecies of injuftice. We have been induced to offer thefe remarks from a belief that thofe who ought to be the patrons and guardians of medical fcience, have fallen into the fame error as Jack did, who tore the lace off his coat with fuch violence that he tore the coat to pieces.

To return to the work before us; the object of which is to recommend a remedy, or rather a medicine and regimen which had never been exhibited in the moft advantageous manner, for the cure of Pthifis, by the Author's predeceffors.

Dr. R. lays no claim to the difcovery of a panacea, or even a fpecific for all poffible cafes of confumption. What he appears to wifh, is to call the attention of the public to the propereft mode of dofing and adminiftering a fubftance which had long been acknowledged as a valuable remedy in affections of the lungs. We have often employed it ourfelves with decided advantage in thofe complaints, in confequence of the recommendation of Dr. Crichton; but we now believe, in dofes too fmall to produce its full effect. Without feeking for explanations in the various acrimonies of the humoral pathology, which is now beeome obfolete, we are compelled by experience to confefs, that fpices, fpirits, ftrong beer, and even animal food, always aggravate the cough in common catarrhs, as well as confumption; and that mucilaginous fubftances, on the contrary, allay or diminifh it. The truth is, that various fluids, taken into the ftomachs of adults, pafs through the lacteals into the circulation, without undergoing that complete animalization which has been fuppofed. How, otherwife, could an infant at the breaft be intoxicated, or purged, by what the mother takes? inftances of which occur daily. If then it be admitted, that the blood itfelf may be materially changed, in its power of irritating and inflaming the lungs, by diet and medicine; and that the irritation produced in the lungs, by the blood, is the caufe of the fuppuration of the tubercules, or the means of perpetuating the ulceration by the inceffance of the cough; it will not be difficult to conceive, that fuch a plan as Dr. R. recommends will generally be found beneficial. It is not, however, to theoretical reafoning that he feems defirous of appealing, though a few obfervations of that kind appear in feveral parts of his pamphlet, but to facts, and the experience of ten years in different parts of Europe.

As it is an object of importance to avoid the impofitions or obviate the ignorance of herb-gatherers, Dr R begins with giving the fynonymes and characters of the lichen from the moft eminent botanifts, with a full defcription of the plant, illuftrated by a coloured plate, and the places where it flourifhes; he then mentions the opinions of a confiderable number of phyficians on the continent, tinent, who have thought highly of its medicinal virtues, both in phthisis and some other diseases, particularly *tabes dorsalis* and *hooping cough.*

As the chief object of Dr. R's pamphlet is to recommend a new and improved manner of employing this lichen, he is minute in his directions for its purification and preparation. His general practice is to prepare an extract or jelly, by boiling ℥vj of the lichen, which has been washed clean in boiling water, in ℔vj of spring water for an hour or more, and then evaporating the strained decoction, with the addition of ℥vj of refined sugar, to the consistence of a syrup or jelly. This he gives either alone, or mixed with milk, syrups, &c. to the quantity of three or four ounces or more daily, which constitutes the medicinal exhibition. He next considers the virtues of this *moss*, when used as an article of diet, which is a very common practice in Iceland; and we find by our papers, that a Russian has lately obtained a patent for making bread of it. Dr. R. seems, in the cure of consumption, to prefer the use of it with chocolate to any other form of food. He concludes this part of his work with a few rules respecting the diet and regimen of consumptive patients; and then commences the history of about twenty cases, selected so as to introduce as much variety as possible. The cases appear to us fair, and several of them seem sufficiently desperate to give the plan a full trial; but all terminate favourably. We should have been better pleased if a few unsuccessful ones had been added, or we had been told that he had never met with a case which did not yield to his method of treatment. The credit notwithstanding of his method of cure will not rest merely on cases contained in his work; other practitioners will soon have recourse to a remedy so simple, so safe, and so pleasant. To give it, however, a fair trial, it should be exhibited *precisely according to his directions*, both as to medicine, diet, and regimen; and there can be no doubt that it will often be tried in cases which are become incurable, and where it will fail of success. But if it saves only *one third* of those who perish annually by phthisis, Dr. R. will merit the warmest gratitude of the public. If his hopes should be confirmed by the experience of others, there can be no doubt that the lichen will become a considerable article of commerce; and we shall be regularly supplied from Iceland itself, where the best kind is produced in great abundance.

The work concludes with a review of several of the means and remedies which have been proposed by others for the cure of consumption, with the author's objections to them. Indeed, their inefficacy alone would be a sufficient objection, if they were merely innocent; but several of them appear to be *injurious* in phthisis.

We join in the author's request to practitioners, that they will communicate the result of their experience, founded upon a candid, fair, and accurate trial of the plan proposed; convinced that it never can injure any patient, and believing that it will always afford relief, even when it cannot effect a cure.

---

*A Compendium of the Veterinary Art; containing an accurate Description of all the Diseases to which the Horse is liable, their Symptoms and Treatment; the Anatomy and Physiology of the Horse's Foot; Observations on the Principles and Practice of Shoeing; on Feeding and Exercise, the Stables, &c. illustrated by Plates; by* JAMES WHITE, *Veterinary Surgeon to his Majesty's First Royal Dragoons.* London. Badcock. 1802.

THOUGH the health of man conftitutes the principal object of our Journal, we do not, on that account, propofe to exclude the anatomy, phyfiology, or treatment, when in a ftate of difeafe of other animals. Nay, fo intimately is the health of human beings connected with that of other animals, that we may affert the perfection of the former to be unattainable without the latter. Dr. Jenner has obferved, how much the domeftication of animals, for the purpofes of luxury, may have contributed to increafe the catalogue of human difeafes; and we may obferve, that the fame domeftication daily contributes, in an eminent degree, to the prevention or cure of difeafes which it cannot be accufed of producing.

The noble animal, the health of which is the object of the work before us, is now become a neceffary as well as a luxury; and we are much pleafed to fee the care of his health fuperintended by regularly educated practitioners.

M. W. appears to have taken a very comprehenfive and proper view of his fubject; and we have no doubt that his book, together with the labours of his cotemporaries, will foon fuperfede, and confign to oblivion, the miferable jargon of the laft century. The plates appear to be well executed, and well calculated to illuftrate the fubject intended.

---

*Cuvier's Lectures on Comparative Anatomy.*

[Concluded from our laft Number.]

HAVING concluded his account of the organs of motion, the author proceeds, in the fecond volume, to the highly interefting fubject of the organs of fenfe, a fubject which exhibits, perhaps of all others, the moft curious, complicated, and recondite refearches of the anatomift.

Among the organs of fenfe, the head, of courfe, comes the firft under confideration; which is compofed of the cranium or bony cafe, for the protection of the brain; and the face, in which are lodged the organs of fight, fmell, and tafte.

The ftudy of this noble organ becomes peculiarly interefting, when, to the confideration of the ftructure and ufes of its feveral parts, is added, that of the proportional fcale of intellect indicated by the relative magnitude and diftribution of the organs in which we have every reafon to believe this quality refides. This ftudy, which to man is fo flattering, and which forms the moft rational bafis for the more fober part of phyfiognomy, appears to have engaged much of the attention of the learned author of this valuable work, who thus expreffes his opinion on this fubject. After relating fome of the obferved facts, " It is not aftonifhing, therefore, that the form of the head, and the proportions of the two parts which compofe it, are indications of the faculties of animals, of their inftinct, of their docility, and, in a word, of all their fenfitive being. This circumftance renders the ftudy of thefe proportions highly important to the naturalift."

If we might any where expect the ingenious author to give the reins to his imagination, and quit the path of fimple defcription which he had hitherto purfued, it would be here, in following up this curious and interefting fubject; but this is not the cafe, *facts* are ftill his principal and almoft his only object, and the reader, who is in fearch of facts, will give the author high credit for his forbearance.

He introduces, in a few words, the meafurements of the different parts of the head that have been made by Camper, Blumenbach, and other anatomifts, who have peculiarly attended to *Cranioſcopy*, which is founded on the following principles:

The two organs which occupy the greateft portion of the face, are thofe of fmell and tafte, and in proportion as thefe are developed, the proportional magnitude of the face, with refpect to the cranium, is increafed; and vice verfa. An extenfive cranium and a fmall face, therefore, indicate a large brain with little developement of the organs of tafte and fmell, while the oppofite proportions point out a brain of fmall volume, with very perfect organs of tafte and fmelling.

The nature of each animal depends, in a great meafure, on the relative energy of each of its functions, and it may be faid to be influenced and governed by thofe fenfations which are the moft powerful.

The brain is the common centre of the nerves, where all perceptions terminate, and it is the inftrument in which the mind combines thofe perceptions, compares them, reflects, and thinks. Animals appear to enjoy the thinking faculty more perfectly, *in proportion as the mafs of the medullary fubftance, which forms their brain, furpaffes that which conftitutes the remainder of their nervous fyftem*, that is to fay, in proportion as the central organ of the fenfes exceeds their external organs.

This proportion lies peculiarly convenient for examination in the cranium and face, as the former contains the whole of the brain, and the latter the organs of the fenfes, efpecially thofe of fmell and tafte, which act with the greateft force on animals, as they govern the two moft commanding paffions of hunger and love.

Man has, of all animals, the largeft cranium and the fmalleft face, that is to fay, as we have before laid down, the central organ of the fenfes exceeds the external organs in greater proportion than in other animals.

One of the means adopted by Camper, in order to fhew this relative proportion, and which is very fimple though not always fufficient, is to meafure the angle which the *facial line* makes with the *bafilar line*, or the bafis of the cranium. The facial line is fup-

 pofed posed to pass along the edge of the incisor teeth, and the most prominent part of the forehead. The basilar line of the cranium, is that which bisects longitudinally a plane passing through the external meatus auditorii on each side, and the inferior edge of the anterior aperture of the nostrils. Hence it follows, that the more the cranium is enlarged, the more will the forehead project forward, and the facial line will form a larger angle with the basilar.

In man, this angle is much larger than in the other mammalia, and the ancients appeared to have laid much stress on the height of the facial line as a characteristic of a noble nature, since, in their representations of gods and heroes, they usually made it quite perpendicular, (that is, at an angle of 90 degrees with the basilar line) or even projecting forward, which is out of all human proportion.

But shall we venture to launch out so far on this hypothesis as to infer that the proportional scale of intellect *in man* is regulated (ceteris paribus) by the height of the facial angle? In different races of the human species we find even more difference in this angle than exists between man and brutes. There are, likewise, two important circumstances which affect the angle of the facial line; *age* is one of them, for an infant will be born with this line at 90°; and, in the same individual, old age will often sink it to 75°; the *depth of the frontal sinusses* is the other; for, as the author observes, in the sarcophaga, or carnivorous animals, in the hog, the elephant, and some others, the extent of the frontal sinusses swell the cranium in front, and consequently elevate the facial line more than the proportion of the brain would require. The following are some of the measurements of this angle given by Camper: European infant, 90°; European adult, 85°; aged European, 75°; adult negro, 70°; young orang-outang, 67°; Sajapou monkey, 65°; horse, 23°; hare, 30°; &c. &c.

However it may flatter the European physiologist to trace the gradual fall of the facial line from himself, through the negro, to the ape, the monkey, the dog, and to the rest of the brute creation, we ought surely to receive with some caution, a system, which (independently of numerous anomalies and exceptions that might be pointed out) places the human native of Africa on so near a level with the brute.

An accurate description of the osteology of the head in man and different animals is subjoined, which properly prepares the reader for the full and comprehensive view of the nervous system, the brain, and the organs of sense, which occupy the greater part of the volume. A remarkably clear and elegant sketch of the general organization of the nervous system, introduces the anatomical description of its several members. In this, the author adduces several arguments to prove the apparent homogeneity of the parts of the nervous system, and hence he infers that the appropriation of particular nerves to the acquirement of determined sensations, is more to be attributed to the accessary circumstances of blood vessels, and the the like, which render thefe nerves fit for thefe individual offices, than to any original difference in their ftructure.

Hence it would follow, that it is only on account of the fituation, and what may be termed the external relations of the optic nerve, that it is peculiarly devoted to the fenfe of feeing, and fo of the reft. It is inconfiftent with the author's plan, however, to purfue thefe ideas at large, and he foon quits them to return to the ftrictly anatomical part of this interefting fubject.

After defcribing the general diftribution of the nerves in man, and other animals, the author devotes feparate chapters to the *five fenfes*, as they are ufually termed, which he gives in the order of the eye, the ear, the touch, the fmell, and the tafte. The defcription of the organs appropriated to each of thefe functions, is very compleat and comprehenfive; to give more than the anatomy of parts would require diftinct treatifes on optics, phonics, &c. entirely foreign from the purpofe of the work; but occafional digreffions into thefe fubjects are introduced, which fhew a familiar acquaintance with the laws of thefe branches of natural philofophy. The anatomy of the organ of hearing is treated of more in detail, than thofe of the other fenfes, on account of the vaft variety of its parts, the intricacy of their diftribution, and the deep refearches of fome of the moft eminent anatomifts to which it has given rife.

Here terminates the fecond volume of this highly valuable, but as yet unfinifhed work. Though incomplete, it is not, however, imperfect, or hereby rendered in any degree unfit for prefent ufe; the anatomical defcriptions are full yet concife, perfectly clear and intelligible (as far as can be done without drawings or reference to particular fpecimens) but not tedious and verbofe; and as thefe volumes fupply, in fo admirable a manner, the want which has long been feverely felt, of a general view of Comparative Anatomy, every anatomift and ftudent of anatomy will feel the higheft obligations to the illuftrious author, and earneftly wifh for a completion of his labours.

The tranflation is uniformly good, and appears to be executed with perfect fidelity. The tranflator has had the advantage of the affiftance and infpection of Mr. Macartney, who, in the prefatory advertifement, has given the reader entire fecurity in all the effential points, by acknowledging himfelf "refponfible for the fidelity of the tranflation as far as refpects the fcience." He has likewife taken confiderable pains to adopt the new French nomenclature of anatomy, employed by the author, to the Latin, and (where it could be done) to the Englifh terms in common ufe, a tafk which confiderably increafes its value to the Englifh reader.

As we hope foon to fee a completion of this excellent work by the learned and eminent author, we truft that when it appears it will fpeedily be given in an Englifh drefs; and we fhall beg leave to fuggeft, that befides a copious index, fome comparative view of the anatomical nomenclature of the refpective countries will then be peculiarly acceptable.

---

*A Treatise on the Morbid Affections of the Knee Joint*; *by* JAMES RUSSELL, *F. R. S. E. Fellow of the Royal College of Surgeons, and one of the Surgeons to the Royal Infirmary of Edinburgh. Edinburgh*, 1802, 8*vo. p.* 242.

DISEASES of the knee joint are juſtly looked upon as ſome of the moſt formidable and threatening maladies that come under the care of the ſurgeon; their diagnoſis is often extremely difficult; the event of a large proportion of them is frequently of the moſt calamitous nature, and from their intimate connection with ſcrophula, the frequency of their occurrence is unfortunately greater in this iſland than in moſt other parts of Europe.

The author of the preſent work, whoſe extenſive practice muſt have afforded him ample opportunity of conſulting perſonal experience, has here given a conciſe view of ſeveral of the moſt ſerious diſeaſes of the knee-joint, with the mode of treatment which he conſiders as the moſt ſucceſsful; and the reader will not be diſappointed in expecting here the ſame perſpicuity and accuracy which diſtinguiſh the Eſſay on Necroſis.

The firſt chapter treats of ſuperficial injuries on this part, which, the author obſerves, on account of the delicacy of the part, are often followed by the moſt dangerous conſequences. Extenſive ſurfaces of tendon and capſular ligament, ſeldom, if ever, granulate kindly, and even a ſlight injury in ſuch parts is always liable to proceed to extenſive inflammation, ſloughing, and a long train of local and conſtitutional evils.

The ſecond chapter notices the injuries upon the burſæ, belonging to the broad tendon which connects the knee with the patella. As this is a part very liable to accidental blows, to injury from kneeling, and the like, it often is the ſeat of tenſe diffuſed fluctuating ſwellings, which may be either the effect of ſuppuration, or of ſimple accumulation of the mucous fluid of the burſæ. Theſe ſhould be diſtinguiſhed, as often the practice is entirely different.

A deſcription of the appearance of tumours, containing blood, is given in the third chapter; which tumours are of more rare occurence, are often very indolent in their progreſs, and when converted into an open ſore, prove exceedingly tedious, painful, and troubleſome of cure.

Having deſcribed thoſe kinds of tumours which might ſometimes be miſtaken for the white ſwelling, the author, in the fourth chapter, gives a detail of the ſymptoms, progreſs, and varieties of this dreadful complaint. After relating theſe, he notices ſeparately the ravages which it occaſions in the different parts in which it is ſeated, ſuch as, morbid effuſion on the capſular ligaments, gradual waſting of the cartilages, and eroſion of the articulating bones. The nature of white ſwelling he decidedly refers to ſcrophula.

A rare anomaly of diſeaſed joint (which is illuſtrated by a plate) is given in the ſeventh chapter. In ſuch caſes, the joint becomes enlarged into a firm ſwelling of conſiderable ſize and irregular ſhape. The progreſs of the diſeaſe is extremely rapid, but the conſtitutional ſymptoms of hectic do not prevail with great ſeverity till till the ſwelling is at its height. On examination, after death, or amputation, the head of the tibia is either extremely enlarged and rendered quite ſpongy or honey-combed in its texture, or it is almoſt wholly conſumed. In either caſe, the bone is very fragile, and even the head of the fibula ſuffers, which is not the caſe in common white ſwelling. At the ſame time, the ſoft parts become gelatinous and of a ſchirrous-like appearance. The author conſiders it as by far the moſt hopeleſs caſe that can occur. If left to itſelf, it proves fatal by the conſtitutional diſeaſe, and if the ſevere meaſure of amputation is reſorted to, the patient (in all the caſes which the author has ſeen) has died of ſubſequent hæmorrhage.

The very painful, though not generally dangerous complaint of moveable bodies within the knee-joint, is next conſidered at ſome length. For the cure, unfortunately, nothing but exciſion can afford any proſpect of ſucceſs, and this operation is at all times ſo hazardous, that it is only adviſeable when the pain and inconvenience of the complaint are intolerable.

The method of cure occupies more than a third of the volume, and the merit of all the uſual remedies is diſcuſſed with conſiderable attention. We do not find much novelty in the mode of cure propoſed: On the firſt attack, leeches and ſaturnine lotions are recommended, where the ſymptoms are evidently inflammatory; but if otherwiſe, aſtringent applications, eſpecially decoction of oak bark and alum. Bliſters are recommended very warmly in every caſe. Among the topical ſtimulants, the author particularizes the powder of gum ammoniac moiſtened with vinegar of ſquills, and the ſavine ointment recommended by Mr. Crowther.

After treating of the cure of white ſwelling, the author conſiders the mode of practice in ſimple inflammation, gouty and rheumatic affection, and ſwellings of the burſæ mucoſæ.

A ſhort chapter on anchyloſis and a few formulæ for ſome of the applications here recommended, conclude the voulme. Three plates are added, one of the diſeaſed enlargement of the head of the tibia deſcribed in the ſeventh chapter, and the two others of different ſpecies of anchyloſis.

---

*Practical Information on the Malignant Scarlet Fever and Sore Throat, in which a new Mode of Treatment is freely communicated*; *by* E. PEART, *M. D.* 1802, pp. 64. 8vo.

As our principal object in this Retroſpect of Medical Publications, is to relate, in a few words, the *facts* that may be brought forward in the numerous publications that are laid before the public, and the practical information to be derived from them, we ſhall only obſerve on the pamphlet before us, that the author, after deſcribing the circumſtances of a very malignant fever and ſore throat that prevailed in the ſummer and autumn of 1801, and reſiſted moſt of the uſual remedies, relates, that he employed a ſolution of ammonia preparata, or carbonate of ammonia, in the proportion of two drachms to five ounces of water, of which two tea-ſpoonfuls were taken every two, three, or four hours, according to the urgency of the ſymptoms.

The ſuccefs, he aſſerts, was ſo great, and ſo much beyond his moſt ſanguine wiſhes, that he conſiders this remedy as almoſt a ſpecific in this dreadful malady. The title of this pamphlet, *Practical Information,* is not very happily choſen, as the above ſimple fact is all the practical matter in the volume. A large portion of it is occupied with a violent attack on the modern chemical theories, very improperly, though commonly, termed *French,* as this term can only be applied, excluſively, to the ſyſtem of nomenclature, and by no means to the diſcovery of the facts, or even the theories which have given it birth. If the manner of our author's attack is not the happieſt, we muſt allow that he has ſelected his adverſary's weak ſide, for ſuch we may certainly conſider the faſhion or rage for explaining by mere chemical principles, and eſpecially by the agency of oxygen, the operation of ſo many of the moſt powerful medicines.

The author takes no little credit to himſelf for divulging the improvements which he perſuades himſelf he has made in the treatment of ſeveral diſorders, *and not making a pecuniary advantage* of them as *secrets.*

---

*Practical Information on Inflammation of the Bowels and strangulated Rupture, in which a new Method of treating those Disorders is faithfully communicated*; by E. Peart, M. D. 1802. pp. 38. 8vo.

This treatiſe is certainly more practical than the laſt. The author, after a few remarks on the nature of enteritis and of incarcerated hernia, gives it as his opinion, that the two complaints are ſimilar in nature, only that the latter has an aſſignable cauſe, which is not always the caſe with the former. After undervaluing in ſome degree the importance of the operation for hernia, on account of its removing only the cauſe of the diſeaſe, and not always its effects, he proceeds to propoſe the new diſcovered remedy for both theſe diſorders, announced in the title page—" And what is it?" the reader will aſk. " Is it digitalis, cold affuſion, or ſome new gum from New Holland?"

This new remedy is a pill compoſed of five grains of calomel, and another of one grain of opium, which are to be repeated every hour or two; ſometimes, however, increaſing the opium if the pain be great, or diminiſhing it, if the patient is eaſy.

Caſes are given, we doubt not with fidelity, in which both hernia and enteritis were removed by this ſimple practice; and the author deſcribes his great ſatisfaction, " at having now; *for the first time,* found a *remedy,*" (we give his own words and italics) " evidently poſſeſſed of prompt and powerful influence over the diſeaſe."

---

*Practical*

*Practical Information on St. Anthony's Fire, or Erysipelas, and on Erythematous Affections in general; as also on the Measles, in which new Modes of Treatment are communicated*; by E. PEART, M. D. 1802. p. 33. 8vo.

THE author introduces the remedy by the hiſtory of a caſe of ſevere eryſipelatous inflammation of the right leg, attended with high fever, delirium, and a very formidable train of ſymptoms. He ſaw the patient after he had been ill for three days, during which time he had undergone the uſual mode of medical treatment, but the fever continued, and the part threatened gangrene. He immediately preſcribed one drachm of volatile alkali to be made into pills, with aromatic confection, to be taken every two or three hours. As an external application, he directed a ſolution of ammonia preparata, ſugar of lead, and ſpirit of ammonia in water; and the precipitated lead was directed not to be waſhed off. The patient recovered ſpeedily under this practice, which, under theſe circumſtances, was certainly very judicious, and probably contributed much to the cure. We have, however, heard of the uſe of ammonia and aromatic confection in ſimilar caſes, before we learnt that it was a diſcovery of Dr. Peart.

The ſame alkali is propoſed in the worſt caſes of meaſles, but more doubtfully.

---

*Anatomical Plates of the Bones and Muscles, diminished from Albinus, for the Use of Students in Anatomy and Artists, and accompanied by explanatory Maps*; by ROBERT HOOPER, M. D. 1802. p. 72. 12mo.

WHEN the magnificent plates of Albinus are cut down to a very ſmall duodecimo, all that can be expected, is to preſerve a general outline and diſtribution of parts.

The plates are as neatly executed as could reaſonably be expected, and if employed as the A, B, C of anatomy, they will have their uſe. After each plate from Albinus, a ſketch for reference is added, and the ſketches or maps are waſhed with different colours in the ſeveral parts, which renders the explanation and reference very eaſy.

This little volume is intended to accompany the author's *Anatomist's Vade Mecum*.

---

*The Anatomy of the Brain, explained in a Series of Engravings*; by CHARLES BELL, *Fellow of the Royal College of Surgeons of Edinburgh*. 1802.

THIS beautiful, accurate, and elegant work is a real acquiſition to the ſtudy of anatomy. The engraving is coloured, and is executed in a ſoft delicate ſtyle, which moſt happily expreſſes the natural appearance of the parts, an advantage of peculiar value in the complicated and minute anatomy of the brain. The author is himſelf the draughtſman, a circumſtance which tends to enſure the fidelity fidelity of repreſentation, and ſtrongly enforces the utility of making the art of drawing an eſſential in the education of thoſe whoſe profeſſion requires a knowledge of the ſituation of natural objects.

This work conſiſts of twelve plates (one of which is copied from the ſplendid and admirable work of the late much lamented Vicq d'Azyr) and the poſition of the different parts is ſo varied, as to give a very clear idea of the anatomy of this noble organ. Peculiar attention appears to be beſtowed on the repreſentation of the ventricles and the parts immediately contiguous to them in the centre of the ſkull.

Each plate is accompanied with its explanation, and the figures of reference are very delicately inſerted in the plate, without injuring the general effect in any ſenſible degree.

An Appendix is added, in which the claim of the preſent Profeſſor of Anatomy at Edinburgh to the diſcovery of the communication between the two lateral ventricles of the brain is oppugned from the writings of Vieuſſens, Winſlow, Haller, and even the older anatomiſts.

